# GC-Derived EVs Enriched with MicroRNA-675-3p Contribute to the MAPK/PD-L1-Mediated Tumor Immune Escape by Targeting CXXC4

**DOI:** 10.1016/j.omtn.2020.08.020

**Published:** 2020-08-21

**Authors:** Ping Li, Xingdong Luo, Yue Xie, Pengfei Li, Fangyong Hu, Junfeng Chu, Xiaojun Chen, Wenbo Song, Ali Wang, Guangyu Tian, Xiang Gu

**Affiliations:** 1 Department of Central Laboratory, Huaian Tumor Hospital & Huaian Hospital of Huaian City, Huaian 223200, P.R. China; 2 Department of General Surgery, Huaian Tumor Hospital & Huaian Hospital of Huaian City, Huaian 223200, P.R. China; 3 Department of Experimental Surgery-Cancer Metastasis, Medical Faculty Mannheim, Ruprecht Karls University, 68167 Mannheim, Germany; 4 Department of General Surgery, Gaoyou Traditional Chinese Medicine Hospital, Gaoyou 225600, P.R. China; 5 Department of Oncology, Jiangdu People’s Hospital Affiliated to Medical College of Yangzhou University, Yangzhou 225200, P.R. China

**Keywords:** gastric cancer, immune escape, extracellular vesicles, microRNA-675-3p, CXXC finger protein 4, programmed cell death 1 ligand 1

## Abstract

MicroRNAs (miRNAs) delivered by gastric cancer (GC)-secreted extracellular vesicles (GC-EVs) are associated with the immune escape in GC. Microarray analysis based on the GEO: GSE112369 dataset identified the presence of poorly expressed CXXC finger protein 4 (CXXC4) in GC, which was validated in clinical samples of GC patients. Moreover, prediction based on TargetScan analysis demonstrated the putative miR-675-3p binding site in the 3′ UTR region of CXXC4. Thereby, our study aims to determine the role of GC-EV-encapsulated miR-675-3p in GC. First, CXXC4 was found to be negatively correlated with programmed cell death 1 ligand 1 (PD-L1). The effects of mitogen-activated protein kinase (MAPK) signaling on GC were evaluated using activator of the MAPK pathway. The overexpression of CXXC4 led to a downregulated MAPK signaling pathway, thus decreasing PD-L1 expression to augment the proliferation and activation of T cells co-cultured with GC HGC-27 cells. GC-EV-encapsulated miR-675-3p negatively regulated the expression of its target gene CXXC4. GC-EV-encapsulated miR-675-3p increased PD-L1 expression to stimulate the immune escape *in vitro* and EV-encapsulated miR-675-3p accelerated cisplatin resistance *in vivo*. Collectively, the aforementioned findings present a mechanism in which EV-mediated miR-675-3p upregulates PD-L1 expression, promoting immune escape in GC.

## Graphical Abstract

**Figure:**
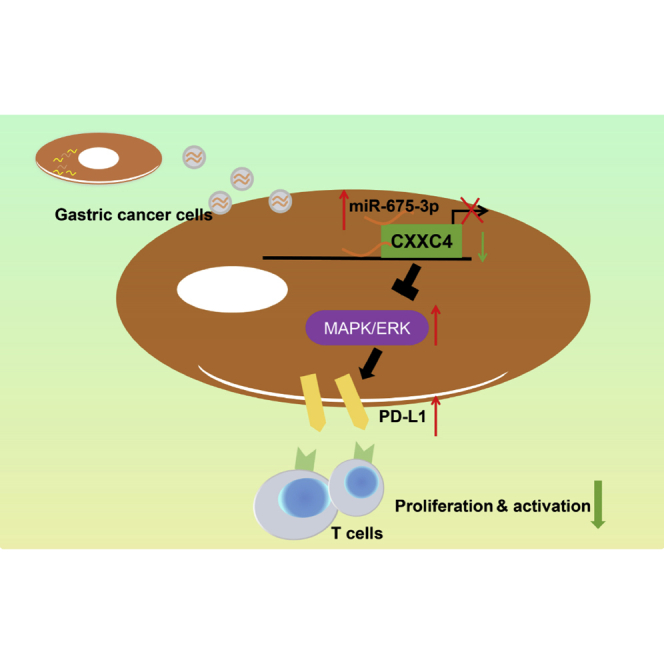

## Teaser

Gastric cancer-derived extracellular vesicles enriched with miR-675-3p inhibit the target gene CXXC4 and elevate PD-L1 expression through the MAPK signaling pathway, thus stimulating the immune escape and cisplatin resistance of gastric cancer cells.

## Introduction

According to the global cancer statistics for 2018, gastric cancer (GC) is the third leading cause of cancer-related death and ranks as the fifth top malignancy in the world.^1^ The underlying factor in mortality reported in GC patients is diagnosis at a later stage or metastasis of the tumors.^2^ The progression of GC is closely related to the immune escape of tumors.^3^ Tumor immune escape is considered as the phenomenon when tumor cells escape from the immune system, which often results in tumor growth.^4^ Hence, a strengthened understanding of the immune response is of great value in the development of novel treatment for cancer. In previous literature, microRNAs (miRNAs) derived from human melanoma extracellular vesicles (EVs) are able to affect the immune response of tumor cells.^5^ Our study was designed to explore the role of GC-EVs enriched with miRNA in the immune escape mechanism of GC.

Multiple cells are capable of releasing EVs, which have been found to be pervasively involved in human cancers, and EVs contained in GC have been reported to mediate GC tumorigenesis and immune evasion.^6^ Meanwhile, EV-loaded miRNAs are proposed as biomarkers for GC.^7^ Based on a previous study, miRNA-675 (miR-675) served as a predictor of poor prognosis of GC, which distinctly accelerates the proliferation of GC cells by binding to paired-like homeodomain transcription factor 1.^8^ Moreover, the EV-encapsulated miR-675 can promote the migration and invasion of cancer cells in metastatic osteosarcoma.^9^ CXXC finger protein 4 (CXXC4) was selected as the subject of the current study, due to the finding obtained from microarray-based analysis that detected the differentially expressed CXXC4 in GC. CXXC4 has been reported to be poorly expressed in patients with GC, and knockdown of CXXC4 stimulates GC cell migration and proliferation.^10^ Furthermore, CXXC4 is considered as a novel tumor suppressor due to its ability to inhibit the mitogen-activated protein kinases (MAPK) signaling pathway,^11^ while the downregulation of the MAPK signaling pathway has been proposed to be capable of reducing the expression of programmed cell death 1 ligand 1 (PD-L1) in lung adenocarcinoma cells.^12^ PD-L1 has been suggested to regulate the immune escape mechanism in human cancers.^13^ Therefore, this study focuses on providing evidence on the role of GC-EVs enriched with miR-675-3p in the tumor immune escape of GC.

## Results

### CXXC4 Is Poorly Expressed and Negatively Correlated with PD-L1 in GC

**Figure 1:**
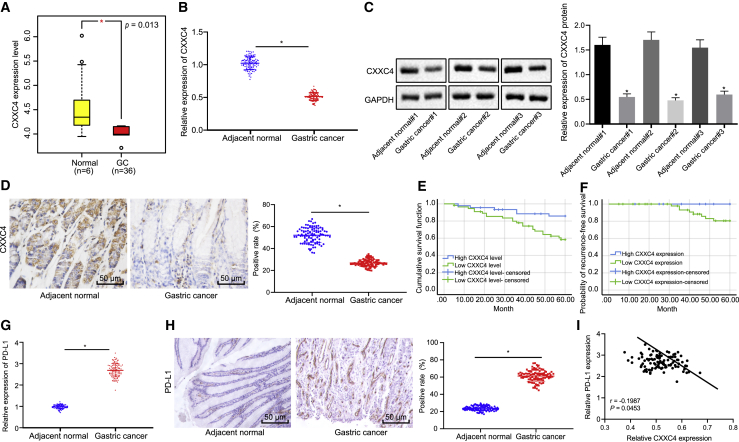
CXXC4 Expression Is Reduced and Negatively Correlated with PD-L1 Expression in GC (A) The expression of CXXC4 in GC samples (n = 36) and normal samples (n = 25) in GEO: GSE112369 from the GEO database. The y axis indicates the gene expression of CXXC4 in microarray GEO: GSE112369. (B) CXXC4 expression in GC tissues and adjacent normal tissues of GC patients (n = 102) detected using qRT-PCR. (C) Expression of CXXC4 normalized to GAPDH in three randomly selected pairs of GC tissue and adjacent normal tissue from patients with GC determined using western blot analysis. Images were quantified using ImageJ software after three biological repetitions. (D) CXXC4 expression identified by IHC. (E) Survival rate of clinical patients. (F) Recurrence rate of clinical patients in follow-up investigation. (G) PD-L1 expression in GC tissues and adjacent normal tissues of patients measured by qRT-PCR. (H) PD-L1 and CXXC4 expression in GC tissues and adjacent normal tissues determined using IHC. (I) Pearson’s correlation coefficient on CXXC4 and PD-L1 correlation. ∗p < 0.05 compared with the adjacent normal tissue. The measurement data are expressed as mean ± standard deviation. A paired t test was used for comparisons between the GC tissues and adjacent normal tissues. The Kaplan-Meier method was used to evaluate the patient’s survival rate with a log-rank test used to compare the survival differences. Data comparison among multiple groups was analyzed by one-way ANOVA, followed by Tukey’s post hoc test. A Pearson’s correlation coefficient was performed for analysis of the correlation between CXXC4 and PD-L1.

GC-related microarray Gene Expression Omnibus (GEO): GSE112369 was obtained from the GEO database (https://www.ncbi.nlm.nih.gov/geo/), revealing a lower expression of CXXC4 in GC compared with that of the normal samples (Figure 1A). In order to further investigate the role of CXXC4 in GC and its effects on immune escape in GC, the expression of CXXC4 was detected in tissues obtained from GC patients with the application of quantitative reverse transcriptase polymerase chain reaction (qRT-PCR) and western blot analysis. The mRNA and protein expression levels of CXXC4 were significantly decreased in GC tissues compared with the adjacent normal tissues (Figures 1B and 1C, p < 0.05). Additionally, immunohistochemistry (IHC) results revealed significantly lower expression of CXXC4 in GC tissues (Figure 1D, p < 0.05). It was suggested that the low expression of CXXC4 may promote the occurrence of GC. To further explore the relationship between CXXC4 and the survival cycle of GC, clinical studies were conducted to determine the relationship between CXXC4 and the prognosis of patients with GC, the findings of which showed that patients with lower levels of CXXC4 (n = 56) had inferior etiology-specific survival compared with those patients with higher expression of CXXC4 (n = 46) (Figure 1E, p < 0.05). Note that none of the patients with high CXXC4 expression (n = 46) had tumor recurrence, while eight of the patients with low CXXC4 expression (n = 56) presented with recurrence after surgery. The 60-month specific recurrence survival rates for patients with high and low expression of CXXC4 were 100% and 86%, respectively (Figure 1F, p < 0.05).

PD-L1, an important protein to promote tumor immune escape, has been reported to be highly expressed in a variety of tumors. The expression of PD-L1 in GC tissues and adjacent normal tissues was analyzed by qRT-PCR and IHC. Based on the results, GC tissues had a higher expression of PD-L1 relative to the adjacent normal tissues (Figures 1G and 1H, p < 0.05). In order to investigate whether PD-L1 was related to CXXC4, we analyzed the correlation between PD-L1 expression and CXXC4 expression in GC tissues by Pearson’s correlation coefficient. The results displayed a negative correlation between CXXC4 expression and PD-L1 expression (Figure 1I, p < 0.05), suggesting that CXXC4 is related to PD-L1 expression in GC.

### CXXC4 Inhibits PD-L1 Expression through the MAPK Signaling Pathway to Promote T Cell Proliferation and Activation

**Figure 2:**
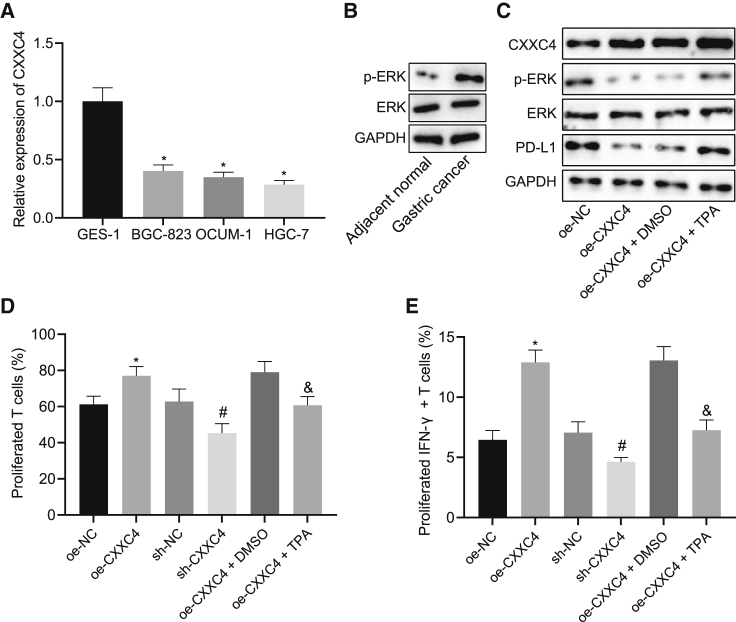
Inhibited PD-L1 Expression Promotes Proliferation and Activation of T Cells (A) qRT-PCR to detect the expression of CXXC4 in human gastric normal epithelial cell line GES-1 and GC cell lines (BGC-823, OCUM-1, and HGC-27). (B) Western blot analysis to detect ERK and phosphorylated ERK content normalized to GAPDH in GC tissues and adjacent normal tissues. (C) The protein expression of CXXC4, PD-L1, ERK, and phosphorylated ERK content in HGC-27 cells determined using western blot analysis after different treatment. (D) Flow cytometry to detect the T cell proliferation after different treatment. (E) Flow cytometry to measure the activation of T cells after different treatment. ∗p < 0.05 compared with GES-1 cells, adjacent normal tissues, or treatment of NC overexpression vector. ^#^p < 0.05 compared with cells treated with sh-NC. ^&^p < 0.05 compared with cells treated with CXXC4 overexpression vector and DMSO. NC, negative control. The measurement data are expressed as mean ± standard deviation. An unpaired t test was used for comparison between the two groups. Data comparison among multiple groups was analyzed by one-way ANOVA, followed by Tukey’s post hoc test. Cell experiments were repeated three times independently.

In order to further study the specific regulatory mechanism of CXXC4 on PD-L1, we conducted qRT-PCR to detect the expression of CXXC4 in the human gastric normal epithelial cell line GES-1 and human GC cell lines BGC-823, OCUM-1 and HGC-27. The results showed that compared with GES-1, the expression of CXXC4 in GC cell lines was significantly reduced, with the lowest expression of CXXC4 observed in HGC-27 cells. Therefore, HGC-27 cells were selected for subsequent analyses (Figure 2A, p < 0.05).

To verify the hypothesis that CXXC4 may regulate the expression of PD-L1 through the MAPK signaling pathway, we examined the expression of extracellular regulated protein kinases (ERKs) and the phosphorylated ERK content in GC tissues and adjacent normal tissues. It was found that the phosphorylated ERK content was higher than that in adjacent normal tissues (Figure 2B, p < 0.05). Subsequently, CXXC4 was overexpressed in HGC-27 cells, followed by detection of the expression of CXXC4, ERK, phosphorylated ERK content, and PD-L1 by western blot analysis. The results showed increased CXXC4 expression in cells treated with CXXC4 overexpression vector, along with decreased phosphorylated ERK content and PD-L1 expression (Figure 2C, p < 0.05). In order to verify the role of the ERK protein in the MAPK signaling pathway, HGC-27 cells received treatment with the ERK activator 12-*O*-tetradecanoylphorbol-13-acetate (TPA) (200 μM) accompanied with the CXXC4 overexpression vector. The phosphorylated ERK content and PD-L1 expression were increased (Figure 2C, p < 0.05).

To investigate the effects of CXXC4-mediated inhibition of PD-L1 on T cell proliferation and activation, T cells were labeled with 5-(and 6)-carboxyfluorescein diacetate, succinimidyl ester (CFSE) and co-cultured with HGC-27 cells after different treatment, followed by the detection of T cell proliferation and the changes in activated interferon (IFN)-γ^+^ T cells as examined by flow cytometry. Moreover, T cell proliferation and IFN-γ^+^ T cell activation were found to be increased secondary to treatment of CXXC4 overexpression vector, yet they decreased in response to silencing of CXXC4. However, the combined treatment of CXXC4 overexpression vector with TPA resulted in attenuated T cell proliferation and IFN-γ^+^ T cell activation (Figures 2D and 2E, p < 0.05). The results indicate that CXXC4 results in the inhibition of PD-L1 expression through the MAPK signaling pathway and then promotes the proliferation and activation of T cells.

### miR-675-3p Targets CXXC4 in GC Cells

**Figure 3:**
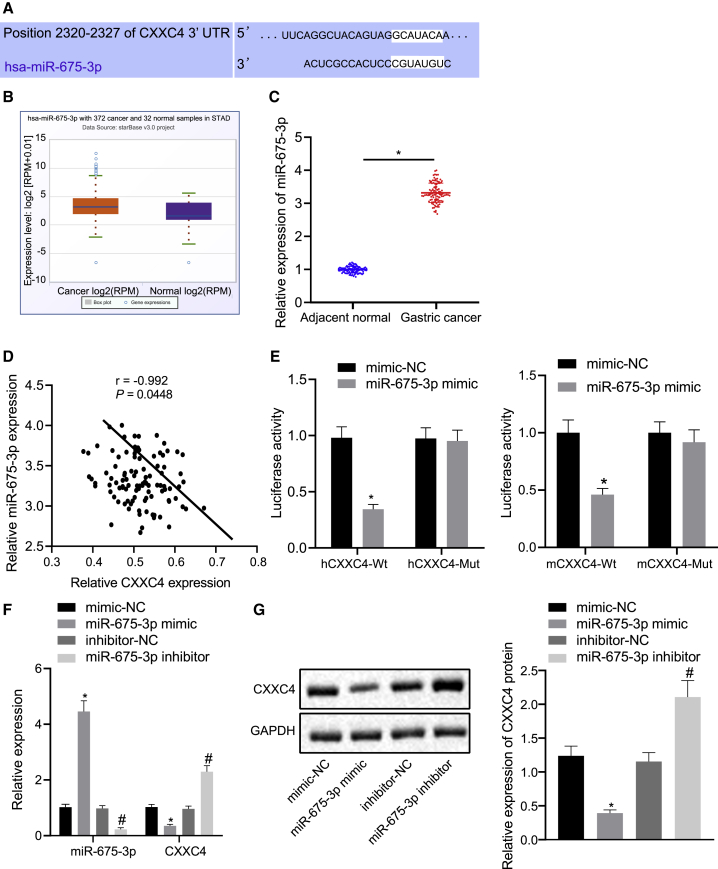
miR-675-3p Specifically Binds to CXXC4 (A) TargetScan prediction on the binding sites between miR-675-3p and CXXC4. (B) Differential expression of miR-675-3p in GC shown in the StarBase database. (C) qRT-PCR detection on miR-675-3p mRNA expression in GC tissues and adjacent normal tissues of GC patients (n = 102). (D) Pearson’s correlation coefficient for the correlation between CXXC4 and miR-675-3p. (E) Dual-luciferase reporter gene assay to verify the targeting relationship between miR-675-3p and CXXC4. (F) qRT-PCR to detect the expression of miR-675-3p and CXXC4 in HGC-27 cells. (G) Western blot analysis to detect the expression of CXXC4 normalized to GAPDH in HGC-27 cells after different treatment. ∗p < 0.05 compared with adjacent normal tissues or cells treated with mimic-NC. ^#^p < 0.05 compared with cells treated with inhibitor-NC. NC, negative control. The measurement data are expressed as mean ± standard deviation. A paired t test was used between the two groups of cancer tissues and adjacent tissues. An unpaired t test was used for comparison between the two groups. Data comparisons among multiple groups were analyzed by one-way ANOVA, followed by Tukey’s post hoc test. A Pearson correlation coefficient was performed for analysis on correlation between CXXC4 and miR-675-3p. Cell experiments were repeated three times independently.

In order to study the upstream regulatory mechanism of CXXC4, we carried out bioinformatics analysis. The TargetScan website predicted that there existed binding sites between miR-675-3p and CXXC4 (Figure 3A). Meanwhile, as the StarBase database predicted, miR-675-3p expression was notably higher in GC tissues than that in adjacent normal tissues (Figure 3B). The expression of miR-675-3p in GC tissues and adjacent normal tissues was evaluated using qRT-PCR, revealing upregulated miR-675-3p expression in GC tissues (Figure 3C, p < 0.05). Pearson’s correlation coefficient was performed to analyze the correlation between miR-675-3p expression and CXXC4 expression, and a negative correlation was found (Figure 3D). The results of a dual-luciferase reporter gene assay verified that CXXC4 (human and mouse) was a target of miR-675-3p. Moreover, the luminescence signal was reduced in cells co-transfected with miR-675-3p mimic and CXXC4-wild-type (WT), suggesting that miR-675-3p could specifically bind to CXXC4 (Figure 3E). The expression of miR-675-3p was further altered in HGC-27 cells, after which CXXC4 expression was detected via qRT-PCR and western blot analysis (Figures 3F and 3G). The mRNA and protein expression levels of CXXC4 were decreased in the cells transfected with miR-675-3p mimic, while the results were reversed following treatment with miR-675-3p inhibitor (p < 0.05). The findings further indicate that miR-675-3p targets CXXC4 in GC cells. Meanwhile, to study the effect of miR-675-3p-mediated inhibition of CXXC4 on GC cells, miR-675-3p was overexpressed in HGC-27 and BGC-823 cells, followed by quantification of CXXC4 expression using qRT-PCR. A Cell Counting Kit-8 (CCK8) assay was performed to evaluate the resultant cell proliferation. It was found that CXXC4 expression was diminished and GC cell proliferation was potentiated in response to overexpression of miR-675-3p (Figure S1).

### miR-675-3p Delivered by EVs Derived from GC Cells Promotes Immune Escape of GC Cells through the CXXC4/MAPK/PD-L1 Axis

**Figure 4:**
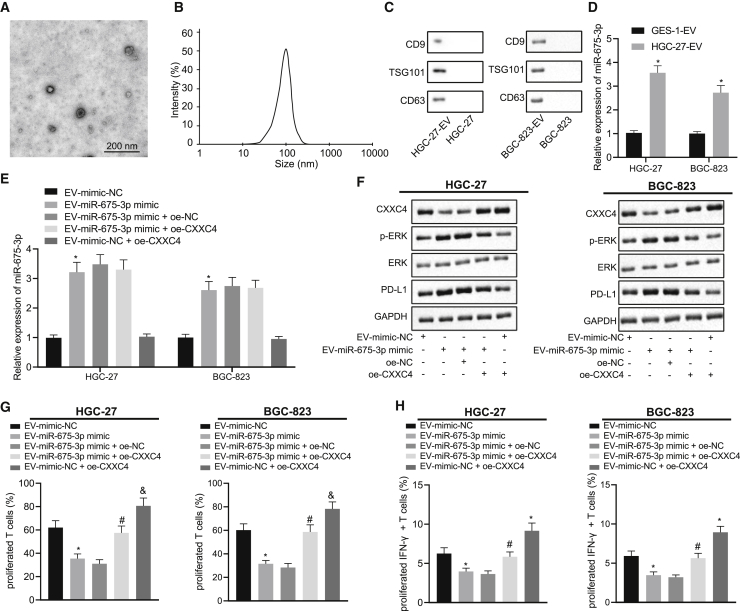
miR-675-3p Delivered by GC-EVs Facilitates the Inhibitory Effect of GC Cells on T Cell Activation, Thus Inducing Immune Escape of GC Cells through the CXXC4/MAPK/PD-L1 Axis miR-675-3p contained in GC-EVs stimulates immune escape in GC cells. (A) Morphological analysis of EVs by TEM (scale bar, 200 nm). (B) Diameter of EVs observed using DLS. (C) The expression of EV surface markers CD9 and CD63 and another EV marker TSG101 in HGC-27 and BGC-823 cell extracts as well as EVs derived from HGC-27 and BGC-823 cells measured using western blot analysis. (D) Expression of miR-675-3p in EVs extracted from GES-1, BGC-823, and HGC-27 cells detected using qRT-PCR. (E) qRT-PCR to detect miR-675-3p expression in HGC-27 and BGC-823 cells after different treatment. (F) Expression of CXXC4, PD-L1, ERK, and phosphorylated ERK content in HGC-27 and BGC-823 cells normalized to GAPDH detected using western blot analysis. (G and H) (G) Flow cytometry was performed to detect T cell proliferation; (H) Flow cytometry was performed to detect the changes in activated IFN-γ+ T cells. ∗p < 0.05 compared with GES-1 cells or cells treated with EV-mimic-NC. ^#^p < 0.05 compared with cells treated EV-miR-675-3p mimic and oe-NC. NC, negative control. The measurement data are expressed as mean ± standard deviation. An unpaired t test was used for comparison between the two groups. Data among multiple groups were analyzed using one-way ANOVA followed by a Tukey’s post hoc test. Cell experiments were repeated three times independently.

In order to determine whether GC-EVs are involved in immune escape, miR-675-3p mimic and inhibitor were transduced into HGC-27 cells, from which the GC-EVs were extracted and analyzed. Under a transmission electron microscope (TEM), the EVs were present in round or oval membrane vesicles (Figure 4A). The size of EVs detected by dynamic light scattering (DLS) ranged from 30 to 120 nm (Figure 4B). The expression of EV surface marker proteins CD9 and CD63 and another EV marker tumor susceptibility gene 101 (TSG101) was determined using western blot analysis, which exhibited that the contents of CD9, CD63, and TSG101 were enhanced (Figure 4C), confirming the successful extraction of EVs (p < 0.05).

The expression of miR-675-3p in EVs derived from the gastric normal epithelial cell line GES-1 and GC cell lines HGC-27 and BGC-823 was detected using qRT-PCR, the results of which displayed that miR-675-3p expression was higher in HGC-27 and BGC-823 cells than that in GES-1 cells (Figure 4D, p < 0.05). The effect of GC-EV-delivered miR-675-3p on immune escape of GC cells was determined by subjecting GC cells to miR-675-3p mimic contained in EVs, after which miR-675-3p, CXXC4, ERK, phosphorylated ERK content, and PD-L1 expression were detected (Figures 4E and 4F, p < 0.05). Within cells treated with EV-miR-675-3p mimic, miR-675-3p expression, phosphorylated ERK content, and PD-L1 expression were increased, while CXXC4 expression was decreased. To further confirm that EV-delivered miR-675-3p affected the expression of PD-L1 through CXXC4/MAPK, CXXC4 was overexpressed simultaneously in GC cells treated with EVs, causing increased CXXC4 expression and reduced phosphorylated ERK content and PD-L1 expression (Figures 4E and 4F, p < 0.05). No significant difference was found regarding miR-675-3p or ERK expression when overexpressed (oe-)CXXC4 was delivered in the presence of EV-mimic-negative control (NC), while CXXC4 was upregulated and PD-L1 expression and phosphorylated ERK content were downregulated.

To investigate the effect of EVs loaded with miR-675-3p overexpression on the proliferation and activation of T cells, T cells were conjugated with CFSE and co-cultured with HGC-27 cells. Flow cytometry was performed to detect T cell proliferation and changes in activated IFN-γ^+^ T cells (Figures 4G and 4H). T cell regeneration was found to be inhibited following treatment with EV-miR-675-3p mimic or EV-miR-675-3p mimic + oe-NC, while EV-miR-675-3p mimic + oe-CXXC4 or EV-mimic-NC + oe-CXXC4 enhanced T cell regeneration. When EVs derived from GES-1 cells were supplemented, T cell proliferation and activation did not differ significantly while they showed significant weakening when EVs derived from HGC-27 cells were added (Figure S2). Moreover, T cell proliferation and IFN-γ^+^ T cells were significantly reduced after T cells were cultured with EV-miR-675-3p mimic. However, the results were opposite after T cells were co-cultured with EV-miR-675-3p mimic and CXXC4 overexpression vector. In the presence of EV-mimic-NC, T cell proliferation and IFN-γ^+^ T cells were also elevated by delivery of oe-CXXC4 (p < 0.05). After co-culture of HGC-27 and T cells, we attempted to explore the involvement of PD-L1 in mediation of T cell proliferation and activation by co-culturing HGC-27 cells with PD-L1 knockout with T cells. We observed that T cell proliferation and activation were suppressed when HGC-27 cells were added into T cells but promoted as a result of the knockout of PD-L1 in HGC-27 cells, indicating that T cell proliferation and activation can be affected by the PD-L1 expression pattern when HGC-27 cells are co-cultured with T cells (Figure S3). These results suggest that miR-675-3p carried by GC-EVs promotes the inhibitory role of GC cells in T cell activation, thus causing accelerated immune escape of GC cells through the CXXC4/MAPK/PD-L1 axis.

### GC-EVs Loaded with miR-675-3p Increase PD-L1 Expression to Promote Immune Escape in GC Cells

**Figure 5:**
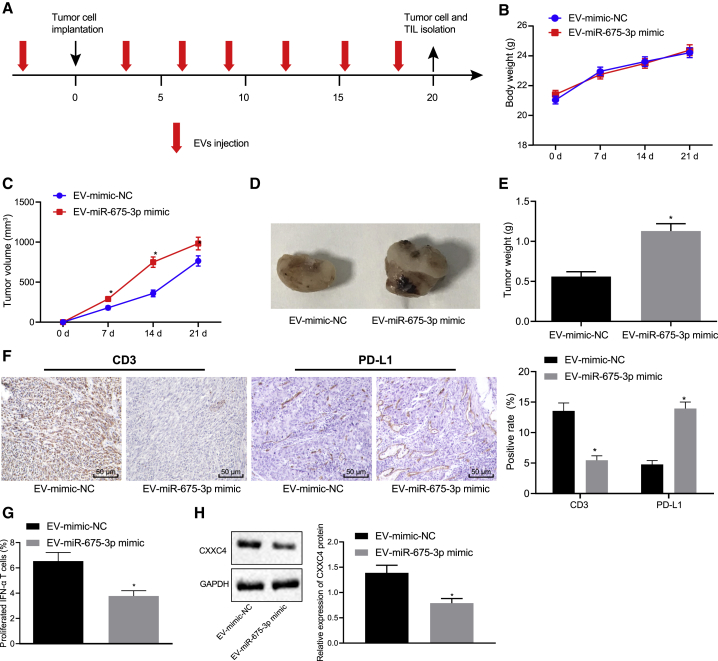
GC-EVs Loaded with miR-675-3p Increase PD-L1 Expression to Stimulate the Immune Escape in Mice Bearing Transplanted Tumors *In Vivo* (A) Process diagram of EV treatment during tumor formation. (B) Quantitative analysis of differences in body weight of mice in each group. (C) Quantitative analysis of tumor volume of mice. (D) Representative images of tumors from mice in each group. (E) Quantitative analysis of tumor weight of mice. (F) IHC assay to detect CD3^+^ expression in T cells and tumors of mice in each group. (G) Flow cytometry to determine the proportion of IFN-γ^+^ in CD3^+^ cells in TILs of tumors. (H) CXXC4 protein level in tumor tissues normalized to GAPDH determined by western blot analysis. ∗p < 0.05 compared with mice treated with EV-mimic-NC. NC, negative control. The measurement data are expressed as mean ± standard deviation. An unpaired t test was used for comparison between the two groups. Data at different time points were analyzed using repeated-measures ANOVA followed by a Bonferroni’s post hoc test. n = 8 in each group.

To investigate the *in vivo* effects of GC-EVs on immune escape, C3H mice were subcutaneously injected with the mouse cell line MFC and subjected to various treatments (Figure 5A). We found that there was no significant difference regarding the weight of the mice in each treatment (Figure 5B, p > 0.05). The tumor growth curve was drawn by measuring the size of the tumors, and the results revealed that the tumors were larger in mice treated with EV-miR-675-3p mimic (Figures 5C and 5D, p < 0.05). A portion of the tumors was sectioned, and the content of PD-L1 in the tumors was detected by IHC. We found that treatment with EV-miR-675-3p mimic led to the enhancement of PD-L1 content in mice (Figure 5E, p < 0.05). Moreover, the expression of CD3^+^ T cells was decreased after the same treatment, as IHC revealed (Figure 5F, p < 0.05). In order to detect the activity of tumor-infiltrating lymphocytes (TILs), T cells in the tumor were isolated, and the difference in the content of IFN-γ^+^ in the T cells was assessed by flow cytometry. The results showed that the IFN-γ^+^ content was decreased in mice treated with EV-miR-675-3p mimic (Figure 5G, p < 0.05). Meanwhile, CXXC4 expression in tumor tissues was determined by western blot analysis, the results of which revealed significantly downregulated CXXC4 expression in the presence of EV-miR-675-3p mimic (Figure 5H, p < 0.05). Taken together, these findings indicate that the immune activity of T cells was weakened following the treatment of EV-miR-675-3p mimic.

### EV-Encapsulated miR-675-3p Reverses the Therapeutic Effects of Cisplatin (DDP) on Mice

**Figure 6:**
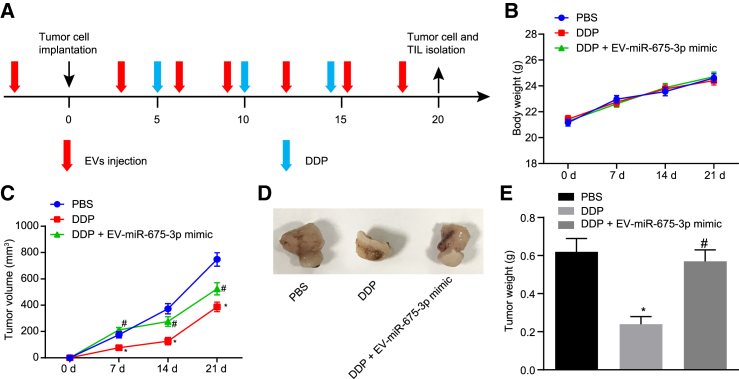
EV-Encapsulated miR-675-3p Increases DDP Resistance in Mice Bearing Transplanted Tumors *In Vivo* (A) Process diagram of EVs and DDP treatment during tumor formation. (B) Quantitative analysis of differences in body weight of mice in each group. (C) Quantitative analysis of tumor volume of mice. (D) Representative images of tumors from mice in each group. (E) Quantitative analysis of tumor weight of mice. ∗p < 0.05 compared with mice treated with PBS. ^#^p < 0.05 compared with mice treated with DDP. The measurement data are expressed as mean ± standard deviation. NC, negative control. Data among multiple groups were analyzed using one-way ANOVA followed by a Tukey’s post hoc test. Data at different time points were analyzed using repeated-measures ANOVA followed by a Bonferroni’s post hoc test. n = 8 in each group.

After determining that EVs loaded with miR-675-3p can promote the immune escape of GC, we then attempted to explore whether EV-encapsulated miR-675-3p was efficient on drug resistance to DDP, a widely used therapeutic agent for GC.^2^^,^^14^^,^^15^ C3H mice were subcutaneously injected with MFC cells followed by various treatments (Figure 6A). There was no significant difference in the tumor weight of mice in each group (Figure 6B, p > 0.05). The tumor weight was measured, showing that the tumors were smaller after the mice received treatment with DDP, suggesting that DDP played an inhibiting role in GC tumor formation. However, treatment of EV-miR-675-3p mimic reversed the inhibitory effects of DDP on tumor growth and subsequently increased the tumor size. Based on these results, EV-encapsulated miR-675-3p could reverse the therapeutic effect of DDP on GC and promote the drug resistance in GC modeled mice (Figures 6C and 6D, p < 0.05).

## Discussion

The immune microenvironment of tumors has significant implications in tumor progression.^16^ Accumulating evidence shows that the EVs derived from tumors are capable of suppressing immune cell function, providing assistance with the immune escape of tumor cells.^17^ Based on a previous report, the development of GC was closely related to tumor immune escape.^3^ In this study, we have investigated the mechanism by which EV-mediated transfer of miR-675-3p is involved in the immune escape of GC, which was implicated in the CXXC4/MAPK/PD-L1 axis.

Initially, the results from our bioinformatics analysis revealed that CXXC4 had an aberrant expression in the GC-related microarray datasets. CXXC4 is considered as a potential suppressor of tumors, which is associated with the prognosis of GC patients in early stages.^18^ The results from our study were further indicative of the presence of poor expression of CXXC4 in GC and that high expression of CXXC4 led to a low tumor recurrence rate. Similarly, altered expression of CXXC4 has been previously implicated in the colon cancer recurrence.^19^ Moreover, we found that CXXC4 expression was negatively correlated with PD-L1 expression. PD-L1 has been demonstrated to stimulate the immune escape of GC cells.^20^ Furthermore, in tumor-infiltrating mast cells, the induction of PD-L1 is able to restrain the proliferation of T cells as well as the production of IFN-γ,^21^ in which the suppression of T cells accelerates the progression of GC.^22^ Subsequently, we found that the regulation of CXXC4 on PD-L1 could be achieved through the MAPK signaling pathway. The MAPK signaling pathway, mediated by mammalian ste20-like kinase 1, is involved in the development of resistance to DDP of GC cells.^23^ Our results found that overexpressed CXXC4 inactivated the MAPK signaling pathway, thus decreasing the expression of PD-L1, which was indicated by detecting levels of ERK proteins and phosphorylated ERK content, which are markers for the MAPK signaling pathway. Consistent with our findings, previous studies have highlighted that CXXC4 is related to the MAPK signaling pathway in hepatocellular carcinoma,^24^ and activation of the MAPK signaling pathway can promote the development of GC.^25^

Next, we focused on the upstream regulatory mechanism underlying CXXC4, and bioinformatics analysis and a dual-luciferase assay were conducted, the results of which indicated that miR-675-3p specifically bound to CXXC4. GC patients have high expression of miR-675.^26^ However, few prior studies show how miR-675-3p derived from GC-EVs functions in GC and the immune escape. In a review of the literature, EV-encapsulated miRNAs were shown to play a promoting role in GC progression.^27^ For instance, EV-encapsulated miR-21 is highly expressed in GC and is taken as a biomarker for the prediction of tumor recurrence in patients with GC.^28^ The evidence derived from our study suggested that miR-675-3p targeted CXXC4. A previous study suggested that miR-629 is able to accelerate the progression of cancer by binding to CXXC4 in colorectal cancer cells,^29^ which further validates our findings that CXXC4 could be a target gene of miRNA. We also evaluated the relationship between miR-675-3p and PD-L1, which showed that GC-EV-delivered miR-675-3p not only promoted PD-L1 expression, but it also suppressed the activation of T cells in GC modeled mice. Similarly, EV-loaded miR-23a-3p upregulates the expression of PD-L1 in liver cancer cells, promoting tumor cell escape.^30^ Note that miRNAs shuttled by EVs have been elucidated to potentiate drug resistance of tumor cells by binding to their intracellular target genes.^2^^,^^14^^,^^15^ DDP is a common therapeutic strategy used in GC, yet DDP resistance can weaken the therapy outcome clinically.^31^ The evidence of our study indicated that GC-EV-encapsulated miR-675-3p could reverse the therapeutic effects of DDP on GC. Moreover, EV-encapsulated miR-675-3p could promote the resistance to DDP in GC modeled mice to advance the GC progression.

In conclusion, based on our *in vivo* and *in vitro* data, our findings suggest that GC-EV-encapsulated miR-675-3p inhibits the expression of the target gene CXXC4 and promotes PD-L1 expression through the MAPK signaling pathway, thus stimulating the immune escape of GC cells. Our study has highlighted a notable role for miR-675-3p delivered by GC-secreted EVs in human GC and identified a novel mechanism of GC-associated T cell-mediated immune escape. Overall, the EV-mediated transfer of the miR-675-3p/CXXC4/MAPK/PD-L1 pathway may be a diagnostic panel for GC. However, additional efforts that will further emphasize the roles and mechanisms of action of EVs in GC are required in order to develop EV-based clinical regimens for GC diagnosis, prognosis, and therapy.

## Materials and Methods

### Ethics Statement

This study was approved by the Ethics Committee of Huaian Tumor Hospital and performed in strict accordance with the principles of the Declaration of Helsinki. All patients participated in this study signed informed consent prior to sample collection. All animal experiments were conducted according to the *Guide for the Care and Use of Laboratory Animals* published by the US National Institutes of Health, with maximum efforts made to minimize animal cruelty.

### Clinical Subjects

A total of 40 pairs of GC and adjacent normal tissues were collected from patients with GC who were hospitalized at Huaian Tumor Hospital during May 2013 to October 2014. Clinical tissue samples were fixed by neutral formalin, paraffin-embedded, and sliced into sections. Meanwhile, we analyzed the clinical records of 102 patients with GC and conducted a 60-month follow-up on postoperative patients to evaluate the survival rate. The detailed information for patients is shown in Table 1. The histopathological evaluation and tumor grading were referred to the standards established by the *WHO Classification of Tumors of the Digestive System.*

**Table 1:** Correlation between CXXC4 Expression and Clinicopathological Features of GC Patients

Clinicopathological Features	Cases (n = 102)	CXXC4 Expression		p Value
		High Expression (n = 46)	Low Expression (n = 56)	
Age (years)				0.163
<50	49	26	23	
≥50	53	20	33	
Sex				0.835
Male	67	31	36	
Female	35	15	20	
Tumor size				0.840
<5 cm	39	17	22	
≥5 cm	63	29	34	
TNM stage				0.041
I + II	64	34	30	
III	38	12	26	

p < 0.05 indicates statistical significance. CXXC4, CXXC finger protein 4; GC, gastric cancer; TNM, tumor, node, metastasis.

### IHC Assay

The paraffin sections of the tissues were deparaffinized by xylene and hydrated with gradient ethanol. Antigen retrieval was performed using citrate acid solution for 1.5 min after boiling. Next, the sections were immersed in 3% H_2_O_2_ (50 μL) for 20 min at room temperature followed by rinsing with phosphate-buffered saline (PBS). Then, the sections were added with rabbit antibodies (Abcam, Cambridge, UK) to CD3 (ab135372), CXXC4 (ab105400), or PD-L1 (ab233482) for overnight incubation at 4°C. The sections underwent further incubation with normal rabbit serum primary antibody used as NC. Afterward, each section was added with 50 μL of polymer reinforcement, incubated at 37°C for 20 min, and then incubated with enzyme-labeled rabbit anti-polymer at 37°C for 30 min. After visualization using diaminobenzidine, the sections were observed under a microscope (CKX53, Olympus, Tokyo, Japan) for 3–10 min. The presence of brown color was regarded as a positive expression. Furthermore, the sections were counterstained by hematoxylin, dehydrated with gradient ethanol, mounted by neutral gum, and observed under a microscope.

### qRT-PCR

Total RNA was extracted from tissues or cells using TRIzol reagent (Invitrogen, Carlsbad, CA, USA), followed by determination of RNA concentration. Primers involved in this study were synthesized by Takara Biomedical Technology (Dalian, Liaoning, China) (Table 2). A miRNA first-strand complementary DNA (cDNA) synthesis (tailing reaction) kit (B532451, Sangon Biotech, Shanghai, China) containing universal PCR primer R was used to obtain the cDNA of miRNA containing a poly(A) tail. The RT of non-miRNA was conducted in accordance with the instructions of a cDNA RT kit (K1622, Reanta Biotech, Beijing, China) and detected in a fluorescence qPCR instrument (ViiA 7, Daan Gene, Guangzhou, China). The relative mRNA transcription level of target gene mRNA was calculated using the relative quantification of the 2^−ΔΔCt^ method with 2 μg of total cDNA as the template and glyceraldehyde-3-phosphate dehydrogenase (GAPDH) or U6 as the internal reference (ΔCt = ΔCt_experimental group_ − ΔCt_control group_, ΔCt = Ct_target gene_ − Ct_internal reference_). The relative transcription level of target genes was determined using the 2^−ΔΔCt^ method, with comparisons made of the gene expressions in each group.

**Table 2:** Primer Sequences for qRT-PCR

	Primer Sequences
miR-675-3p	F: 5′-TGGTGCGGAGAGGGCCCACAGTG-3′
	R: 5′-TGGTGTCGTGGAGTCG-3′
CXXC4	F: 5′-CTCATCAACTGTGGCGTCTG-3′
	R: 5′-TTAGTTTGCCCTTCATTTCC-3′
PD-L1	F: 5′-TGGCATTTGCTGAACGCATTT-3′
	R: 5′-TGCAGCCAGGTCTAATTGTTTT-3′
U6	F: 5′-CTCGCTTCGGCA GCA CA-3′
	R: 5′-AACGCTTCACGAATTTGCGT-3′
GAPDH	F: 5′-GCACCGTCAAGGCTGAGAAC-3′
	R: 5′-ATGGTGGTGAAGACGCCAGT-3′

qRT-PCR, quantitative reverse transcriptase polymerase chain reaction; miR-675-3p, microRNA-675-3p; CXXC4, CXXC finger protein 4; PD-L1, programmed cell death 1 ligand 1; GAPDH, glyceraldehyde-3-phosphate dehydrogenase; F, forward; R, reverse.

### Western Blot Analysis

GC tissues or cells were collected, and the total protein was extracted by radioimmunoprecipitation assay (RIPA) lysis buffer (Beyotime Biotechnology, Shanghai, China). The protein concentration was determined using a bicinchoninic acid (BCA) kit (20201ES76, Yeasen Biotechnology, Shanghai, China). Quantification was performed according to different concentrations. Following separation with the use of polyacrylamide gel electrophoresis, the protein was transferred onto a polyvinylidene fluoride membrane (IPVH85R, Millipore, Darmstadt, Germany), which was then blocked with 5% bovine serum albumin (BSA) for 1 h at room temperature. Next, the membrane was incubated with primary rabbit antibodies (Abcam, Cambridge, UK) to CXXC4 (ab105400, 1:100), ERK (ab184699, 1:10,000), phosphorylated ERK (ab79483, 1:1,000), PD-L1 (ab233482, 1:100), and GAPDH (ab128915, 1:10,000) overnight at 4°C. The following day, the membrane was cultured with horseradish peroxidase (HRP)-labeled goat anti-rabbit immunoglobulin G (IgG) (ab205718, 1:20,000, Abcam, Cambridge, UK) for 1 h at room temperature. The membrane was visualized using the developing solution. Protein quantitative analysis was conducted using ImageJ 1.48u software (National Institutes of Health) and expressed as the gray value ratio of each protein to the internal reference GAPDH.

### Cell Culture and Transfection

HEK293T cells were purchased from the Cell Bank of the Chinese Academy of Sciences (Shanghai, China). Human gastric normal epithelial cell line GES-1 and GC cell lines BGC-823 and OCUM-1 were purchased from CoBioer Biosciences (Nanjing, Jiangsu, China). The mouse GC MFC cells were obtained from Procell Life Science & Technology (Wuhan, Hubei, China). All cells were cultured in Dulbecco’s modified Eagle’s medium (DMEM) (12800017, Gibco-BRL, Gaithersburg, MD, USA) containing 1.5 g/L NaHCO_3_, 10% fetal bovine serum (FBS), and 1% penicillin-streptomycin.

HGC-27 cells in logarithmic growth phase were seeded in a six-well plate at a density of 4 × 10^5^ cells/well. Confluent cells were transfected based on the instructions of Lipofectamine 2000 reagents (11668-019, Invitrogen). HGC-27 cells were transfected with CXXC4 overexpression vector, short hairpin RNA (shRNA) targeting CXXC4 (sh-CXXC4), miR-675-3p mimic plasmid, miR-675-3p inhibitor plasmid, knockout plasmid of PD-L1 (ko-PD-L1), or their corresponding NCs. The transfection sequences and plasmids were purchased from GenePharma (Shanghai, China) as shown in Table 3. The CXXC4 overexpression vector was the pmirGLO vector.

**Table 3:** Sequences for Cell Transfection

RNA	Forward Sequence	Reverse Sequence
miR-675-3p mimic	5′-CTGTATGCCCTCACCGCTCA-3′	5′-TGAGCGGTGAGGGCATACAG-3′
NC mimic	5′-TTCTCTAGCCGTGTCAGCTAA-3′	5′-TAGGTGACACGTTCGGAGAATT-3′
miR-675-3p inhibitor	5′-TGAGCGGTGAGGGCATACAG-3′	
NC inhibitor	5′-CCTAAAGGTZCTCCTGATCA-3′	

miR, microRNA; NC, negative control.

As for the MAPK signaling pathway, the HGC-27 cells were treated with 200 μM activator TPA for the ERK pathway, with dimethyl sulfoxide (DMSO) as the NC.

### CCK8 Assay

Cells in each group were seeded in a 96-well plate at a density of 1 × 10^3^ cells/well and cultured in 100 μL of medium containing 10% FBS for 1–5 days. The number of cells was then measured with reference to the manual of CCK8 (Dojindo Molecular Technologies, Japan) by adding 10 μL of CCK8 solution in each well for a 1-h incubation. An optical density (OD) value at 450 nm was determined by a microplate reader.

### T Lymphocyte Isolation and Identification

Fresh human peripheral blood was collected and the heparin anticoagulant was centrifuged at 1,500 rpm for 5 min, followed by the removal of upper plasma. The lymphocytes were separated by lymphocyte isolation solution and cultured in Roswell Park Memorial Institute (RPMI) 1640 culture medium. Cells were then added in a six-well plate coated with anti-CD3/anti-CD28 tetramer antibody (STEMCELL Technologies) and cultured at 37°C. Cells were re-suspended by sterile PBS and counted. The phycoerythrin (PE)-CD3^+^ (130-113-129, 1:50, Miltenyi Biotec) cells were sorted with the application of flow cytometry.^32^

### Flow Cytometry

Cells were made into a single-cell suspension and re-suspended in staining buffer (BD Biosciences, San Jose, CA, USA). PE-CD3 and permeated Pacific blue-IFN-γ (BioLegend, #505817, 1:50 ratio) were added into T cells, which were detected by a BD FACSCanto II flow cytometer (BD Biosciences) and analyzed by FlowJo software.

### T Cell Proliferation Assay

A 96-well plate was coated with anti-CD3/anti-CD28 tetramer antibody (STEMCELL Technologies). Subsequently, interleukin-2 (20 IU/mL) was added to the CD3^+^ T cells collected, which were labeled by CFSE (S1076, Beijing Solarbio Science & Technology, Beijing, China) and co-cultured with GC cells in coated plates of RPMI 1640 medium at 37°C and 5% CO_2_ for 5 days. Flow cytometry was used to measure the differences of CFSE content.^32^

### Isolation and Identification of GC Cell-Derived EVs

GES-1 or HGC-27 cells were cultured in EV-free DMEM containing 20% FBS. After 48 h, the medium was collected and centrifugation was carried out at 5,500 rpm for 15 min for the removal of cells and cell debris. EVs were extracted from the supernatant following the instructions of the ExoQuick-TC kit (EXOTC10A-1, Shanghai Shanran Biotechnology, Shanghai, China). EVs were precipitated, fixed in 2.5% glutaraldehyde at 4°C, dehydrated with gradient alcohol, and embedded in epoxy resin. Sections were stained with uranyl acetate and citrate acid lead, observed, and imaged under a TEM (JEM-1010, JEOL, Tokyo, Japan).

EV particles were dissolved in RIPA buffer and quantified using a BCA protein analysis kit (Thermo Fisher Scientific, Rockford, IL, USA). The antibodies (Abcam, Cambridge, UK) used in western blot analysis were as follows: TSG101 (ab125011, 1:1,000), CD63 (ab134045, 1:1,000), and CD9 (ab92726, 1:2,000).

The diameter of EVs was measured using DLS, in which the Zetasizer Nano-ZS90 instrument (Malvern Panalytical, Malvern, UK) was used to activate the light at a wavelength (λ) of 532 nm. EV samples were diluted with 0.15 M NaCl to the appropriate optical signal detection level (ratio of 1:50).

### Dual-Luciferase Reporter Gene Assay

The dual-luciferase reporter gene vector for the 3′ untranslated region of CXXC4 (human and mouse) and mutant plasmids of the mutation sites binding to miR-675-3p were established as pmirGLO-hCXXC4-WT, pmirGLO-hCXXC4 mutant type (MUT), pmirGLO-mCXXC4-WT, and pmirGLO-mCXXC4-MUT. The reporter plasmid miR-675-3p mimic and the relevant NC were co-transfected into HEK293T cells, respectively. Cells were lysed 24 h after transfection, followed by centrifugation at 12,000 rpm for 1 min, with the supernatant collected. The luminescent signal was detected by the Dual-Luciferase reporter assay system (E1910, Promega). Each cell sample was added with 100 μL of firefly luciferase working solution to detect firefly luciferase, while 100 μL of Renilla luciferase working solution was added to detect Renilla luciferase. The relative luminescent signal was expressed as the ratio of firefly luciferase to Renilla luciferase, with Renilla luciferase as the internal reference.

### Tumor Formation Experiment in C3H Mice

A total of 32 C3H mice (aged 5–7 weeks, weighing 18–22 g) were purchased from Lingchang Biotech (Shanghai, China). The MFC cells in the logarithmic growth phase were resuspended in 200 μL of DMEM solution at the density of 2 × 10^6^ cells/well and injected into the flank of mice. The mice were then injected with or without MFC cells containing 100 μg/100 μL of EV-miR-675-3p mimic, 1,010 mg/kg DDP, and the corresponding NC through the caudal vein 3 days prior to tumor formation. After cell injection, EVs were injected into mice every 3 days, and mice were simultaneously treated with DDP at day 5, 10, and 15.

After tumor formation, tumor size and body weight of mice were measured every 3 days. The volume of tumor was calculated with the formula as (length × width^2^)2. All tumors were extracted 20 days after tumor formation, after which tumor volume, size, and weight were compared. TILs were prepared using a tumor dissociation kit (Miltenyi Biotec, Bergisch Gladbach, Germany) and a TIL gentleMACS dissociator (Miltenyi Biotec). Briefly, tumor fragments were placed in a C tube containing 2.35 mL of RPMI 1640 medium, 10 μL of enzyme D, 50 μL of enzyme R, and 12.5 μL of enzyme A, followed by m_imptumor_03 running. With the removal of the supernatant, the precipitate was rinsed by 10 mL of RPMI 1640 medium, followed by re-suspension to isolate TILs. The gradient Percoll II solution (GE Healthcare) was prepared. TILs were separated from the tumor suspension by density gradient centrifugation and analyzed by flow cytometry. Tumor tissues that were found without the separation of TILs were paraffin-embedded, sectioned, and subjected to IHC assay to detect the expression of PD-L1 (#64988, Cell Signaling Technology, Danvers, MA, USA, rabbit, 1:200) and CD3 (ab22378, Abcam, 1:200 ratio).

### Bioinformatics Analysis

GC-related microarray dataset GEO: GSE112369 was downloaded from the GEO database (https://www.ncbi.nlm.nih.gov/geo/), consisting of 36 GC samples and 6 normal samples, followed by differential expression analysis using the limma package of R language.

### Statistical Analysis

The statistical data were analyzed by SPSS 21.0 statistical software (IBM, Armonk, NY, USA). Measurement data were expressed as mean ± standard deviation. A paired t test was used for comparisons between two groups of paired data that conformed to normal distribution and homogeneous variance, while unpaired data were analyzed using an unpaired t test. One-way analysis of variance (ANOVA) was performed for comparisons among multiple groups followed by Tukey’s *post hoc* test. Tumor volume at different time points was analyzed using repeated-measures ANOVA followed by Bonferroni’s post hoc test. OD values at different time points were analyzed by two-way ANOVA. The survival rates of patients were measured using the Kaplan-Meier method, while the survival difference was detected using a log-rank test. Correlation among miR-675-3p, CXXC4, and miR-675-3p was analyzed by Pearson’s correlation coefficient. A value of p <0.05 indicated a statistically significant difference.

## Author Contributions

Ping Li, F.H., and W.S. designed the study. Y.X., X.C., G.T., and X.G. collated the data, carried out data analyses, and produced the initial draft of the manuscript. Pingfei Li, J.C., and A.W. contributed to drafting the manuscript. X.L. edited and revised the manuscript. All authors have read and approved the final submitted manuscript

## Conflicts of Interest

The authors declare no competing interests.

## Supplemental Information

Document S1. Figures S1–S3

Document S2. Article plus Supplemental Information
