# Supplementary material for: GC-Derived EVs Enriched with MicroRNA-675-3p Contribute to the MAPK/PD-L1-Mediated Tumor Immune Escape by Targeting CXXC4
Source: Mol Ther Nucleic Acids. 2020 Aug 21;22:615–26. doi: 10.1016/j.omtn.2020.08.020 (PMC7578556; doi:10.1016/j.omtn.2020.08.020)
Supplement: Document S1. Figures S1–S3 [file mmc1.pdf]

OMTN, Volume *22*

Supplemental Information

GC-Derived EVs Enriched with MicroRNA-675-3p Contribute to the MAPK/PD-L1-Mediated Tumor Immune Escape by Targeting CXXC4

Ping Li, Xingdong Luo, Yue Xie, Pengfei Li, Fangyong Hu, Junfeng Chu, Xiaojun Chen, Wenbo Song, Ali Wang, Guangyu Tian, and Xiang Gu

Gastric cancer-derived extracellular vesicles enriched with microRNA-675-3p contribute to the MAPK/PD-L1-mediated tumor immune escape by targeting CXXC4

Running title: Effects of EVs loaded with miR-675-3p on GC

Ping Li [1, 2, 3, #], **Xingdong Luo** [2, #], **Yue Xie** [4, #], **Pengfei Li** [1], **Fangyong Hu** [1], **Junfeng Chu** [5], **Xiaojun Chen** [5], **Wenbo Song** [5], **Ali Wang** [5], **Guangyu Tian** [5], **Xiang Gu** [5, *]

[1] Department of Central Laboratory, Huaian Tumor Hospital & Huaian Hospital of Huaian City, Huaian 223200, P.R. China

[2] Department of General Surgery, Huaian Tumor Hospital & Huaian Hospital of Huaian City, Huaian 223200, P.R. China

[3] Department of Experimental Surgery-Cancer Metastasis, Medical Faculty Mannheim, Ruprecht Karls University, Mannheim 68167, Germany

[4] Department of General Surgery, Gaoyou Traditional Chinese Medicine Hospital, Gaoyou 225600, P.R. China

[5] Department of Oncology, Jiangdu People's Hospital Affiliated to Medical College of Yangzhou University, Yangzhou 225200, P.R. China

[#] These authors contributed equally to this work.

[*] **Corresponding author: Xiang Gu**, MM, Department of Oncology, Jiangdu People's Hospital Affiliated to Medical College of Yangzhou University, No. 9, Dongfanghong Road, Yangzhou 225200, Jiangsu Province, P.R. China

E-mail: 734909944@qq.com

Tel.: +86-13813130311

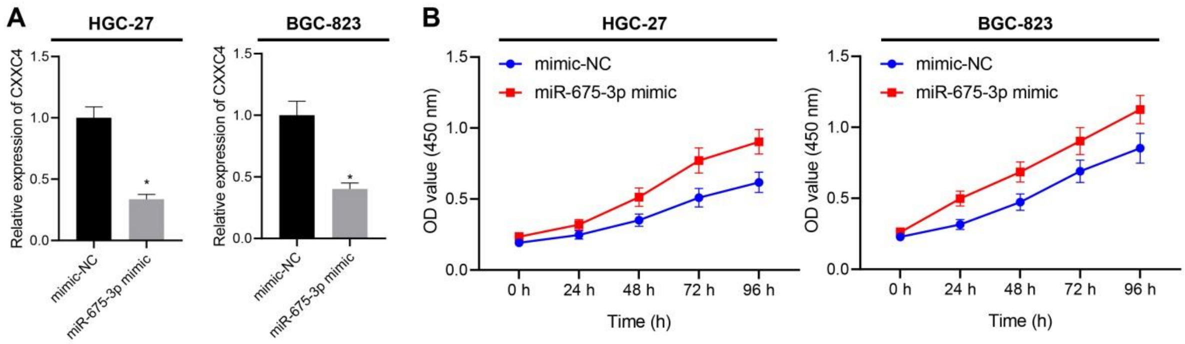

Figure S1 Effect of miR-675-3p-mediated inhibition of CXXC4 on GC cell proliferation. A, Expression of CXXC4 in HGC-27 and BGC-823 cells determined by RT-qPCR. B, HGC-27 and BGC-823 cell proliferation detected by CCK8 assay. The measurement data are expressed as mean ± standard deviation. Unpaired *t*-test is used for comparison between the two groups. Data at different time points are analyzed using two-way ANOVA.

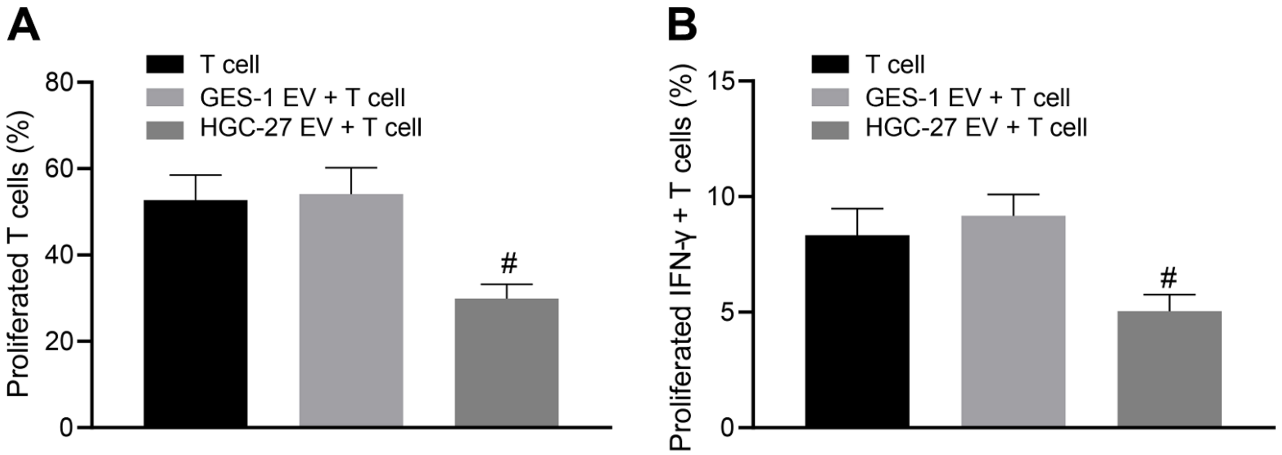

Figure S2 Effect of EVs derived from different cells on T cell proliferation and activation. A, T cell proliferation detected by flow cytometry in the presence of EVs derived from different cells. B, T cell activation detected by flow cytometry in the presence of EVs derived from different cells. The measurement data are expressed as mean ± standard deviation. Data among multiple groups are analyzed using one-way ANOVA followed by Tukey's post hoc test. Cell experiment is repeated 3 times independently.

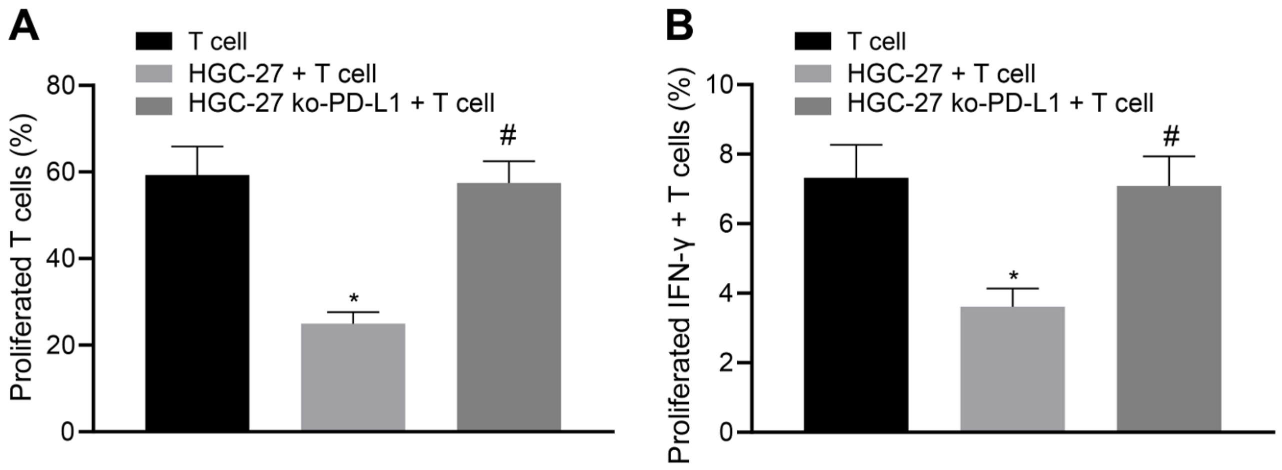

Figure S3 T cell proliferation in HGC-7 cells is dependent on PD-L1. A, T cell proliferation detected by flow cytometry in the presence/absence of GC HGC-27 cells with/without PD-L1 knockout. B, T cell activation detected by flow cytometry in the presence/absence of GC HGC-27 cells with/without PD-L1 knockout. The measurement data are expressed as mean ± standard deviation. Data among multiple groups are analyzed using one-way ANOVA followed by Tukey's post hoc test. Cell experiment is repeated 3 times independently.
